# *Eichhornia crassipes* (Mart.) Solms (natural or carbonized) as biosorbent to remove pollutants in water

**DOI:** 10.1007/s42452-021-04736-9

**Published:** 2021-07-23

**Authors:** Herbert de P. Lima, Yvan J. O. Asencios

**Affiliations:** Institute of Marine Sciences, Federal University of São Paulo (UNIFESP), Maria Máximo St. 168, Santos, SP 11030‑100 Brazil

**Keywords:** Biosorption, Dyes, Heavy metals, *Eichhornia crassipes*, Carbon, Microphyte

## Abstract

The prolific aquatic herb *Eichhornia crassipes* considered a pest in many countries can cause damage such as obstruction of water flows and impair the locomotion of fishing boats. However, *E. crassipes* is renewable, inexpensive, and widely available in nature, and its ability to adsorb recalcitrant pollutants with mutagenic and carcinogenic properties, including synthetic dyes and heavy metals, has been extensively studied by the scientific community. This review paper analyzes previous reports concerning the use of *E. crassipes* (in the natural and carbonized form) as an adsorbent for heavy metal cations and textile dye. The adsorptive capacity of *E. crassipes,* the best conditions (adsorbent dosage, pH, and temperature) for the removal of these pollutants, the mechanism of adsorption, and the comparison between natural and carbonized forms (advantages and disadvantages) are discussed. All the results revised in this review indicated that the use of *E. crassipes* (and its carbon derived) as adsorbent is promising and is an excellent material to be applied in the water treatment. It could be used in the actual technologies for the treatment of contaminated water by heavy metals and textile dyes; however, more studies need to be made on scale-up, economy projects, and related issues, to be finally implemented in wastewater treatment plants.

## Introduction

**Fig. 1:**
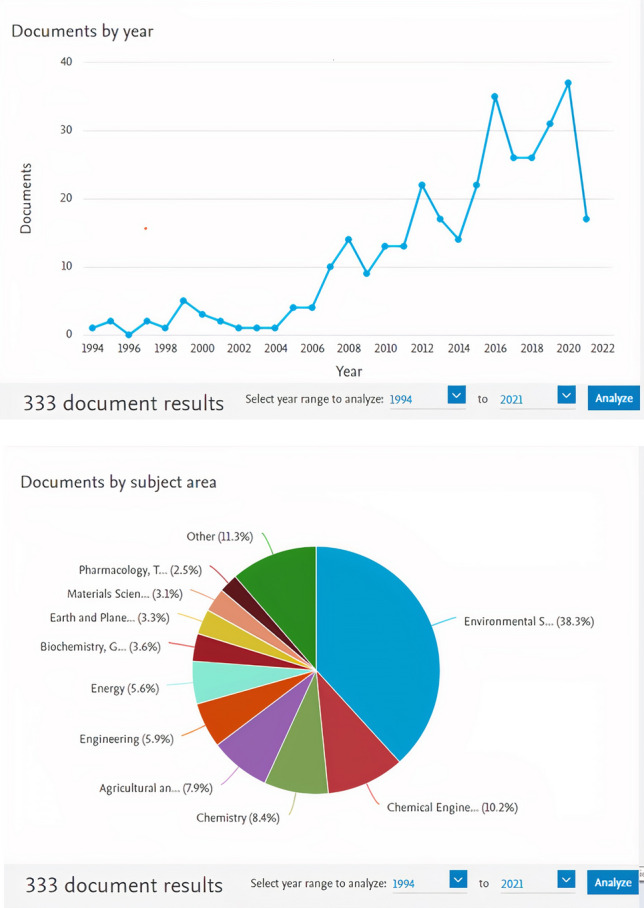
Results extracted from Scopus database

*Eichhornia crassipes* is a prolific aquatic macrophyte, and owing to its bioaccumulation property (the accumulation of pollutant substances or other chemicals in the organisms), it is used as an indicator of pollution (to indicate the presence of nutrients such as nitrogen and phosphorus in eutrophic water bodies). The presence of *E. crassipes* can obstruct the flow of water in channels, dams, and rivers; the damage caused by its prolific can also affect the livelihood of surrounding communities. However, because *E. crassipes* is renewable, inexpensive, and widely available in nature, its ability to adsorb recalcitrant pollutants with mutagenic and carcinogenic properties (including synthetic dyes and heavy metals), has been extensively studied by the scientific community. For instance, a simple search in the Scopus database [1], with the keywords: “*E. crassipes*” and “biosorption” reported that 333 documents were published since 1994 (88% corresponded to original articles), the major part of these documents corresponds to the field of environmental science, followed by chemical engineering and chemistry, and these results are shown in Fig. 1.

Biosorption is a physicochemical process that occurs spontaneously when dead biomass adsorbs pollutants (from water) on its cellular structure. In the case of *E. crassipes* the wall cell is composed principally of *Lignin*, *Hemicellulose*, and *Cellulose* (among others elements in minor proportion) providing functional groups such as: –NH_2_ (amines), –COOH (carboxyls), –OH (hydroxyls), –SH (sulfhydryl), –C=O (carbonyls), involved in the removal of pollutants from water. Biosorption can be reversible, and the intensity of the interaction (among biomass and pollutants) will depend on the chemical affinities between the pollutants (molecules or ions) and the surface organic groups present on the cell wall of the dead biomass (through different mechanisms like complexation, coordination, chelation, ion exchange, or adsorption).

In biosorption, the dead biomass is used instead of living biomass (as occurs in bioaccumulation, where the absorption rate of a substance is faster than that at which the substance is lost by catabolism and excretion). As biosorption derives from the adsorption concept (adsorption on a solid surface, dominated by physical and chemical forces), several advantages are obtained such as recyclability (allowing regeneration), high rates of pollutant removal (after optimization), and scale-up feasibility; among these, it is possible to apply chemical treatments aiming to improve the adsorptive properties.

The use of *E. crassipes* as biosorbent in water treatment systems (aiming to produce drinking water) can help to reach goal 6 of the Sustainable Development Goals (17 SDG, established by ONU) that refers to “clear water and sanitation.” Moreover, the use of *E. crassipes* as biosorbent is under the Green Chemistry Principles, and the principles concerning the Less Hazardous Chemical Synthesis, Use of Renewable Feedstock, and Design for Degradation, are covered because such microphyte is not toxic, is eco-friendly, renewable, and biodegradable [2].

Given the highlights mentioned in the previous paragraphs, this review paper analyzes some previous reports concerning the use of *E. crassipes* (in the natural or carbonized form) as an adsorbent for heavy metal cations and textile dye. The adsorptive capacity of *E. crassipes*, the best conditions (adsorbent dosage, pH, and temperature) for the removal of these pollutants, the mechanism of adsorption, and the comparison between natural and carbonized forms (advantages and disadvantages) are discussed. All the results revised in this review indicated that it could be used in the actual technologies for the treatment of contaminated water by heavy metals and textile dyes; however, according to our analysis, more studies need to be made on scale-up, economic projects, and related issues, to be finally implemented in wastewater treatment plants. This review paper is divided into the following parts: general aspects of contamination caused by dyes and heavy metal cations; classification of synthetic dyes; treatment methods for wastewater; details of *E. crassipes*; *E. crassipes* and its carbon derived as biosorbents for synthetic dyes and heavy metal cations, and finally conclusions and future perspectives.

## Contamination by synthetic dyes and heavy metals in water

Water is an indispensable natural resource for the health of the population and the sustainable development of society [3]. However, the discharge of liquid effluents resulting from activities involved in the production of the most varied consumer goods has been one of the biggest environmental pollution problems faced by the contemporary world [4]. The deposition of recalcitrant substances in water represents a series of risks to the integrity of the environment and the health of living beings [5]. Among these substances, we can highlight the synthetic dyes, widely used in the production of paper, printing inks, cosmetics, medicaments, food, and textiles [4–6]. These industries consume large amounts of water and produce high volumes of colored effluents that can contribute to the contamination of the aquatic systems [7].

Currently, there are about 100 000 varieties of synthetic dyes available on the market, with approximately 7 × 10^5^ tons produced annually by industries specialized in the manufacture of those products [8]. It is estimated that during industrial coloration processes, approximately 10–15% of the world's production of these dyes is dumped into water bodies [9]. The molecular structures of synthetic dyes are designed to be resistant to the most diverse environmental conditions [10]. Therefore, the accumulation of those colored substances in the water generates several negative impacts on the aquatic ecosystems, such as reduced photosynthetic rate, decreased oxygenation of the aquatic environment, inhibition of the growth of local biota, carcinogenic, mutagenic, and teratogenic effects in some fish species [8, 11, 12].

Another aggravating factor is related to the presence of toxic substances with carcinogenic properties, such as Cd, Cu, Pb, Zn, and Co, present in the chemical constitution of many of these dyes [12]. Much attention has been focused on industries of the textile sector, as they are often responsible for the discharge of large amounts of colored liquid effluents near rivers, lakes, and seas [13, 14]. A large number of scientific articles, literary reviews, and published studies on the removal of dyes in aqueous solutions using biosorption techniques can be considered a reflection of global concern about issues related to water pollution [4]. The rivers can transport these pollutants and contribute significantly to the increase in the pollution levels on the beaches [15].

### Synthetic of dyes

The absorption of light by dyes occurs through atomic groups called chromophores that often have heteroatoms like N, O, and S with non-binding electrons in their structure [4]. The classification of dyes, based on their chemical structure, is related to the type of chromophore present in the molecule, as seen in Table 1 [5]. Besides the chromophores, groups called auxochromes are introduced into the aromatic rings of dyes, whose main function is to fix the dyes in the materials [16]. Some of the main auxochromatic groups are: –NH_2_, –NHR, –NR_2_, –OH, –OCH_3_, among others [16]. Dyes are also classified according to the application mode: basic, acid, direct, reactive (all anionic), and dispersed (non-ionic), taking into account their load on dissolution in an aqueous medium [5].

**Table 1:**
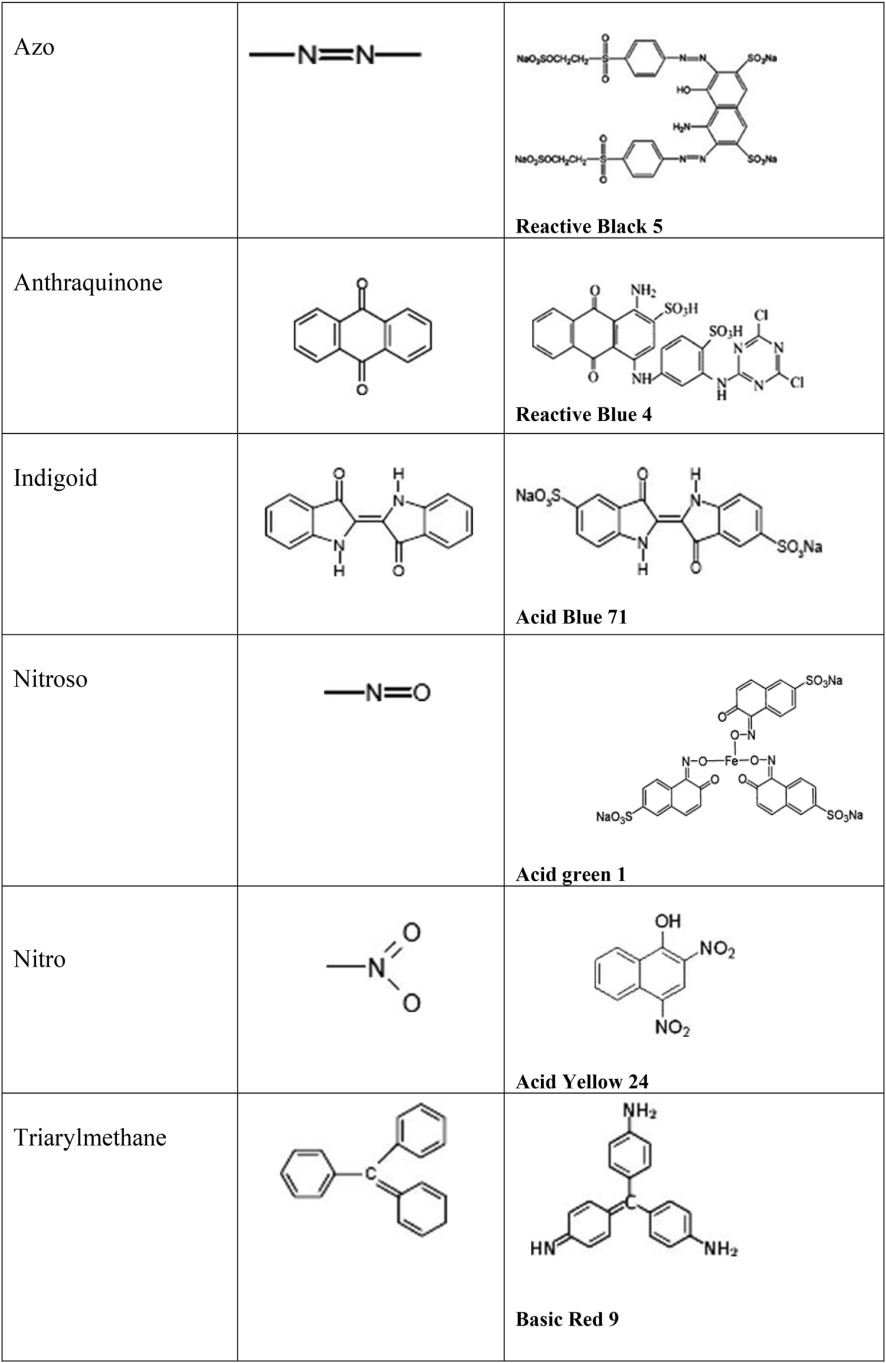
Classification of dyes according to their chemical structure. *Source*: adapted from [5]

### Treatment methods for contaminated water by dyes

**Fig. 2:**
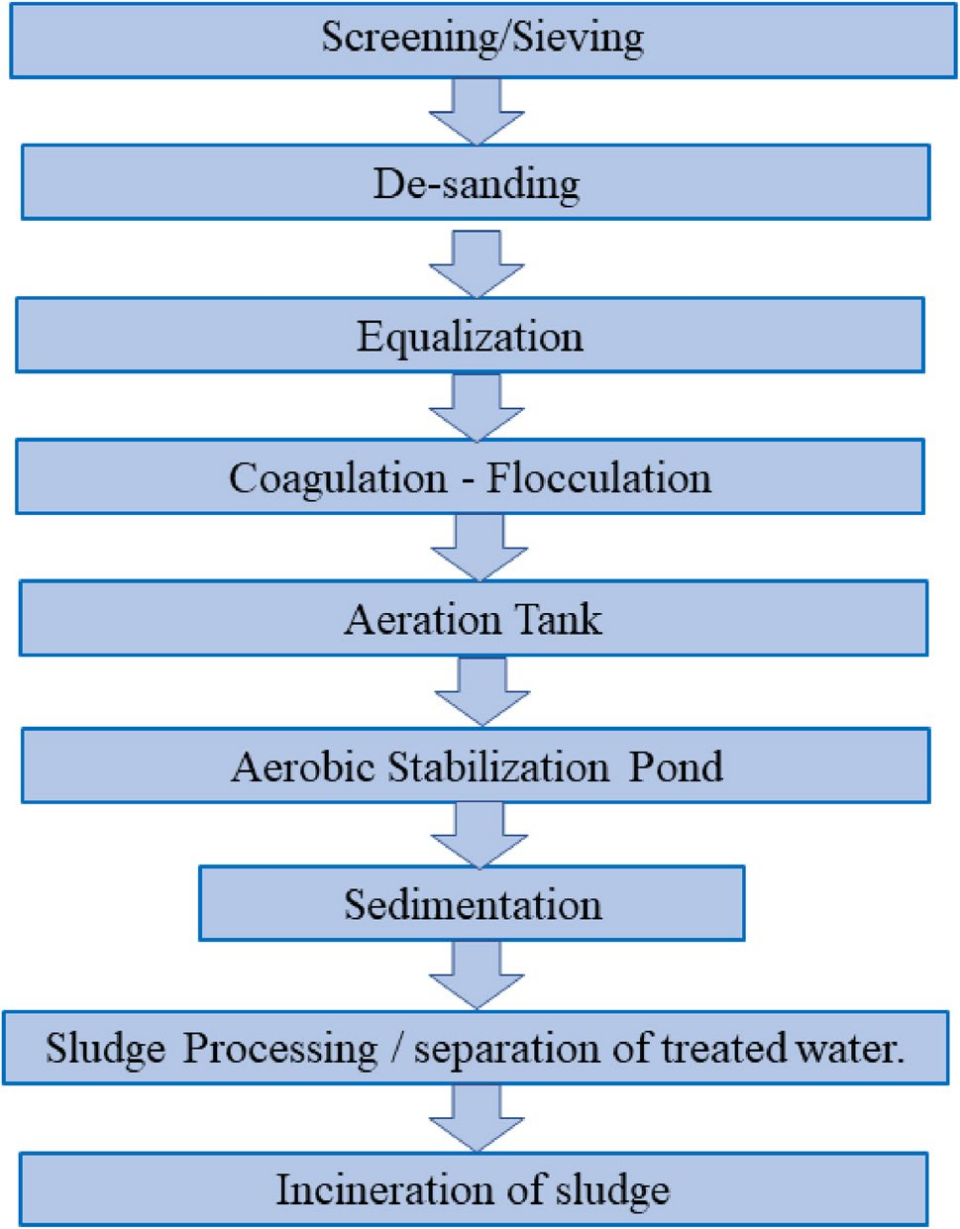
The traditional wastewater treatment system in the textile industry (adapted from [17])

Common water treatment does not remove the synthetic dyes at the level that is required to avoid pollution. However, the industries adopt specific setups aiming to diminish the pollution caused by synthetic dyes and to fulfill the limits established by legislation. Figure 2 shows the wastewater setup followed by some textile industries. The order can vary from one textile industry to another, but in general, the major part of such treatments is included in this figure (adapted from Ramos et al. 2020.)

In Fig. 2, the process screening/sieving refers to coarse solids removal present in the effluent. De-sanding involves the removal of sandy particles with a density greater than 2 g cm^−3^. Equalization consists to homogenize the effluent that will be treated, in an aerated tank. In coagulation–flocculation, the destabilization of colloids occurs by coagulating agents and consequent formation of flakes. The aeration tank consists of a biological reactor for the removal of dissolved organic matter and part of the dyes. In the aerobic stabilization pond, the pond contains microorganisms that have action in the removal of biodegradable organic matter. Sedimentation serves to separate matter (flocs, sedimentable particles, biological sludge) by the difference of density. In sludge processing, the moisture of sludge is reduced, since sludge is usually disposed in industrial landfills. Finally, in some cases, the dry sludge is incinerated.

The setup shown in Fig. 2 is known as the traditional dye removal method; due to its high cost of operation and maintenance, it was stopped in some countries [17]. Nowadays, much research is being done to develop more advanced methods, so that dye wastewater can be reused. Several treatments based on biological, chemical, and physical processes are studied to remove synthetic dyes from wastewater [18–20]. Although these treatments have some advantages, in some cases they can generate secondary products (that in some case needs further treatments) and compromise their viability for large-scale operations [19]. Some advantages and disadvantages of these treatments can be seen in Table 2.

**Table 2:** Advantages and disadvantages of dye removal methods in water (adapted from [14, 21, 22])

Methods	Advantages	Disadvantages
*Physical treatments*		
Membrane filtration	Removes all types of dyes	Produces concentrated sludge
Ion exchanges	Regeneration does not compromise the adsorbent	Not effective in removing all dyes
Coagulation / flocculation	Economically viable	High sludge generation
Adsorption with Activated carbon	Removes a wide variety of dyes	Very high cost
*Chemical treatments*		
Oxidation	Short reaction time and efficient for dye degradation	Difficulty in activating the H_2_O_2_, an effective removal depends on the catalyst
Ozonation	Can be applied in a gaseous phase, effective in removing dyes, and does not generate sludge	A short half-life (20 min), can generate toxic by-products
Photochemical degradation	Does not produce sludge, reduced odors	Generation of toxic by-products in some cases
Electrochemical degradation	Does not consume chemicals, does not generate sludge	High flow rates cause a direct decrease in dye removal
*Biological treatments*		
Anaerobic systems dye bioremediation	Allow the removal of azo dyes and other water-soluble dyes	Produces methane and hydrogen sulfite by anaerobic digestion
Adsorption on microbial biomass	Some dyes have a particular affinity to bind with microbiological species	Not effective in removing all dyes
Mixed microbial cultures	Maximum time of 30 h for decolorization of wastewater (relatively fast)	Removes a limited amount of dyes, high cost for large scale applications
Degradation by algae	Removes dyes, low cost, environment-friendly	Unstable system

Activated carbon has a great adsorption capacity and is one of the most important adsorbents used in physical treatments for the removal of dyes and other pollutants in wastewater; however, the high cost of production and difficulty of regeneration are some of the major problems regarding its application [5]. Other physical methods, like membrane filtration, demonstrate advantages for presenting low energy consumption and easy operational control; however, its main disadvantages are associated with sludge generation and shortened lifetime due to excessive residue accumulation [5, 19, 23]. Chemical methods which include ozonation, electrochemical, and oxidative processes are also employed in contaminated water treatments; however, some of the biggest obstacles associated with the application of these treatments are the high cost of chemical reagents, high demand for electrical energy, and in some case exists the formation of toxic by-products [5, 24]. Biological methods using microorganisms in aerobic or anaerobic degradation processes to remove waste and organic substances present in water, are more economically viable and have easier applicability than physicochemical methods [12, 25]. Biological treatments can degrade a variety of organic molecules; however, the complex structure of organo-synthetic origin of some dyes, such as azo, is not completely degraded under aerobic conditions employing microbiological methods [5, 19, 24].

Although there are some exceptions, most water treatments based on adsorption processes have shown greater viability when compared to other treatments due to their more affordable prices, easy maintenance, flexibility, a wide range of adsorbent options, and absence of harmful substances production [5, 18]. In this way, different adsorbent materials of mineral or biological origins, such as bentonites, zeolites, ash, chitosan, rice husks, corn cobs, among others, have been studied to develop economically advantageous technologies, less aggressive to the environment and effective in removing chemical species incorporated into water, such as metal ions and dyes [13, 24, 26].

Based on this, biosorption has been widely investigated as it shows advantages over conventional methods of treatment, such as minimization of chemical sludge containing waste, the possibility of regeneration, and recovery of biosorbents [27].

### Treatment of wastewater contaminated with heavy metals

**Fig. 3:**
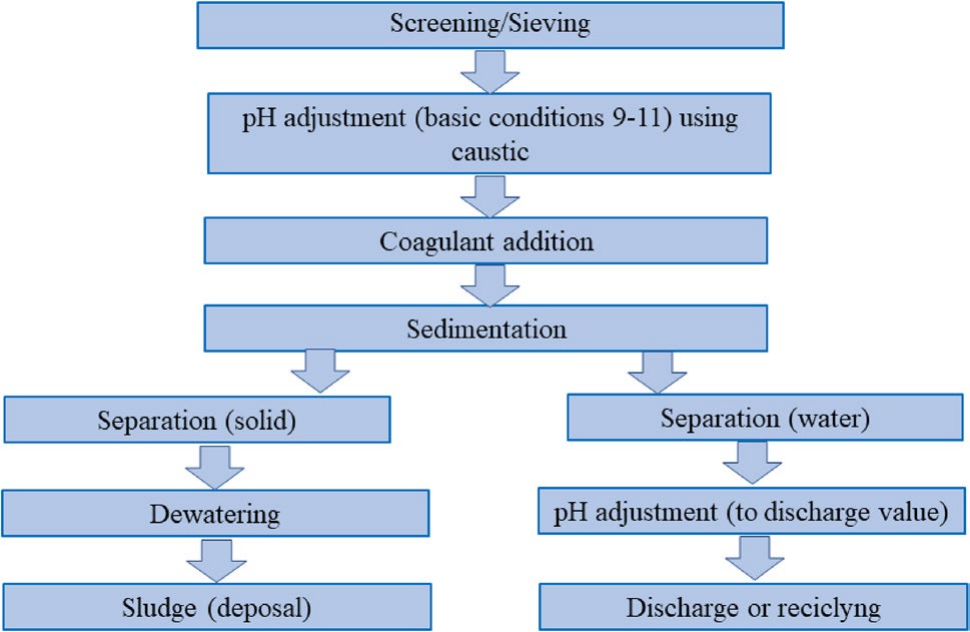
Processes of a conventional metals precipitation treatment plant ( adapted from Barakat et al. [28])

Heavy metal removal from wastewater can be carried out by conventional treatment processes such as chemical precipitation method, electrochemical process, and ion exchange process, and their principal advantages and disadvantages are summarized in Table 3. The chemical precipitation method is the most traditional process for heavy metal removal from wastewater, and their principal steps are resumed in Fig. 3.

**Table 3:** Advantages and disadvantages of heavy metal removal methods water (adapted from [29, 30])

Method	Advantages	Disadvantages
Chemical precipitation	Inexpensive	Sludge is produced at a large amount
	Efficient	
	Simple	
Coagulation–flocculation	Sludge settling	High cost
	Dewatering	Consumption of chemicals in large amount
Ion-exchange	Materials can be regenerated	Efficient at low concentration
	Metal selective	Metal selective (Less number of metal ions can be removed) Fouling and contamination
Electrochemical methods	Metal selective	High cost for implementation
	Chemicals are not necessary	High operation cost
	Pure metals can be obtained	
Adsorption with activated carbon	A wide variety of metals removed	High cost related to activated carbon
	high efficiency	Difficult of regeneration
Adsorption with natural zeolites	A wide variety of metals removed	Low efficiency
	Relatively less cost of materials	
	Possibility of regeneration	
Membrane filtration	Less solid waste produced	High cost related to membranes
	Less chemical consumption	Low quantity of effluent treated
	High efficiency and fast	Removal capacity is affected by the presence of other metals
	Metal selective	Cost related to regeneration and maintenance
	Possibility of regeneration	Fouling and contamination

Chemical precipitation is a very important process for the removal of heavy metals, and it depends on the solubility of the metal involved, concentration (metal ions and relevant anions), and pH of the wastewater. It is considered an inexpensive method as it uses caustic salts (lime or limestone) as the precipitant agent (since is widely available and economic, in many countries) [28]. Its main drawback is the large amount of toxic sludge produced during the treatment, which leads to disposal problems and further treatment requirements.

The remediation by coagulation–flocculation occurs by electrostatic interaction between heavy metal cations and coagulant–flocculant agents. It is often used for wastewater treatment containing heavy metals, and it is highly efficient. However, the main drawbacks of this process are the production of sludge (which contains metal hydroxides, and other toxic compounds) and the expenditure of chemicals.

The ion exchange process is a very useful process to remove heavy metal cations from water, and it uses resins (organic synthesized) which make this process practical. It is considered a relatively low-cost process, and among other benefits it works in room operation conditions. The ion exchange process works well particularly for treating water with a low concentration of heavy metals. The spent resins can be regenerated. However, its main drawback is the limited variety of metals that can be removed, and this process is sensible to the pH of the wastewater and often presents problems related to fouling (for example calcium sulfate), organic matter deposited, and bacterial contaminations [30].

The electrochemical treatments of wastewater containing heavy metal cations can be classified into electro-deposition, electrocoagulation, electro-flotation, and electro-oxidation. Electrocoagulation is the most common method used to heavy metal precipitation (forming coagulants by electrolytic oxidation). The advantages of this process are that relatively less amount of sludge is produced, no chemical is needed, and its ease of operation. The main drawbacks are energy consumption, sludge production (containing aggregates of hydroxide metal precipitates), and slow metal precipitation rate [31].

Membrane filtration is capable of removing suspended solids, organics, and inorganic contaminants including heavy metals in water. Various types of membrane filtration can be applied ultrafiltration (membrane pore size 5–20 nm), nanofiltration, and reverse osmosis. These processes use to be fast and work well in various conditions; for instance, ultrafiltration can reach more than 90% of removal efficiency with a metal concentration ranging from 10 to 112 mg L^−1^ at pH ranging from 5 to 9.5 and at 2–5 bar of pressure. Higher selectivity of separation is expected [30].

### Biosorption

Biosorption can be defined as the ability of biological material to remove pollutants from wastewater through physical–chemical mechanisms present in its structure [27]. Some of the advantages related to the use of biomaterials in the treatment of contaminated water are the good performance to remove contaminants, low cost, relative abundance in nature, and efficiency compared to materials such as, for example, ion exchange resins [32]. Among the biological materials tested as adsorbents can be mentioned: peat, seaweed, chitosan, lignin, fungi, yeasts, and bacteria, among others [33].

However, the dead biomass demonstrates an adsorption capacity equal to or higher than the living biomass, does not require a supply of nutrients, has easy regeneration, and can be stored for long periods at room temperature without the risk of deterioration [25, 32]. The contaminants present in aqueous media are removed by different mechanisms that can occur in different parts of its cellular structure like complexation, coordination, chelation, ion exchange, or adsorption [34]. According to [35], the biosorption process is associated with important functional groups present in biomolecules, as shown in Table 4.

**Table 4:** Some of the main functional groups involved in biosorption. *Source*: adapted from [35]

Structural formulas	Binding groups	Ligand atoms	Occurrence in biomolecules
–OH	Hydroxyl	O	Polysaccharides, uronic acids, sulfated, amino acids
	Carbonyl (ketone)	O	Peptide bond
	Carboxyl	O	Uronic acids, amino acids
–SH	Sulfhydryl (thiol)	S	Amino acids
	Sulfonate	O	Sulfated
–C–S–C–	Thioether	S	Amino acids
–NH_2_	Primary Amine	N	Chitosan, amino acids
NH	Secondary amine	N	Peptidoglycan, peptide bond
	Amide	N	Amino acids
C=NH	Imine	N	Amino acids
	Imidazole	N	Amino acids
	Phosphonate	O	Phospholipids
	Phosphodiester	O	Uronic acids, lipopolysaccharides, teichoic acid

Aquatic macrophytes have been distinguished from other groups of plants due to their great capacity for phytoremediation [36]. Genera of hydrophytes that include *Lemna, Pistia, Azolla,* and *Eichornia,* have considerable potential for the capture and retention of toxic substances like heavy metals [36, 37]. Among these hydrophytes, the aquatic herb *E. crassipes* has a good performance in the adsorption of heavy metals such as Ni, Zn, Cd, Pb, Cr, and Cu, in aqueous solutions, carried out by its dried biomass or its respective ash [38, 39]. However, the removal of heavy metals by these plants varies according to the surface properties of the biosorbent, metal concentration, pH of the aqueous medium, and heavy metal-ion species present in the solution [39]. The high reproduction rate, high pollution tolerance, and the great adsorption capacity of chemical species, such as heavy metals, qualify *E. crassipes* as an alternative and promising biosorbent [40]. In this way, several authors have suggested the possibility of the utilization of biosorbents based on biomass of *E. crassipes* on a large scale for the treatment and control of contaminants like heavy metal and dyes from wastewater generated by various industries [39–41].

#### Eichhornia crassipes (Mart.) Solms

**Fig. 4:**
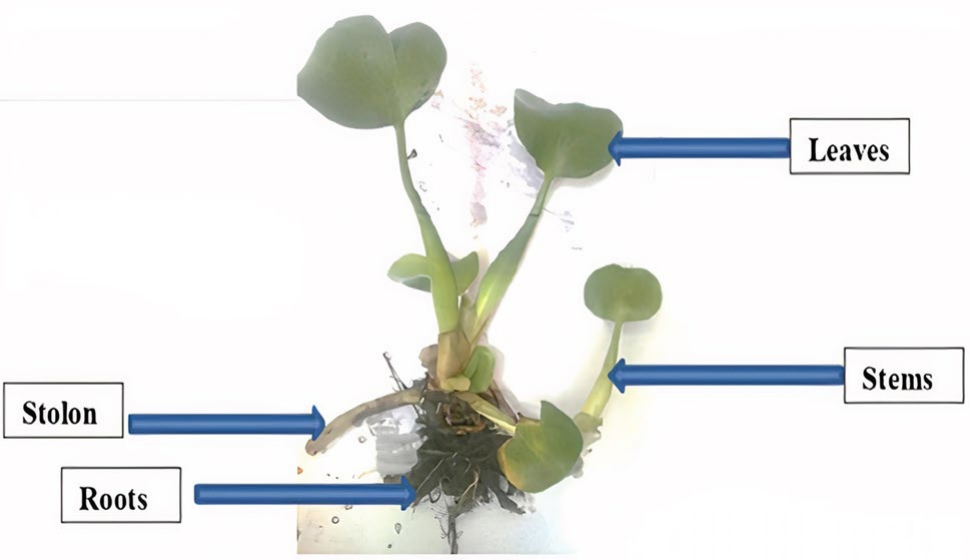
*Eichhornia crassipes* (Mart.) Solms ( *Source*: the Authors)

Several authors have reported the biosorption potential of *E. crassipes* showing promising results [36–44]. *E. crassipes* is a free-floating and extremely prolific aquatic macrophyte, originally from the Amazon Basin (South America) and belonging to the *Pontederiaceae* family [42, 43]. The species was initially described by von Maltus as *Pontederia* in 1823 and was transferred posteriorly to the genus *Eichornia* by Solms Laubach in 1883 [44]. Its reproduction can occur asexually by vegetative organs denominated stolons or sexually by seeds [45]. The leaves of *E. crassipes* have margins ranging from ovate to orbicular, apex obtuse or rounded, and base cuneate or cordate [44]. Other characteristics of the plant are the presence of inflated stems (filled with aerenchyma), stolons (organs directly related to vegetative reproduction), and long adventitious roots [46]. Its flowers have a lilac or purplish color with a yellow spot surrounded by a purple blotch that stands out in the upper median tepal of the perianth [41]. The external morphology of *E. crassipes* can be seen in Fig. 4.

Its deliberate growth is mainly related to the abundant presence of nutrients, such as nitrogen and phosphorus in eutrophic freshwater bodies and with the absence of natural enemies like the grasshopper *Cornops aquaticum* [47, 48]. The presence of *E. crassipes* at high densities can obstruct and reduce the flow of water in channels, dams, and rivers; compromise the locomotion of fishing boats; serve as breeding grounds for disease-spreading organisms; decrease oxygenation in water bodies and interfere with the operation of hydroelectric turbines [49]. The damage and inconvenience caused by this aquatic herb can also contribute to the increase of socioeconomic problems in communities that depend on water for their livelihood [50]. However, *E. crassipes* can be used for activities that include removing contaminants from liquid effluents, biogas production, and cattle feed [51].

Like lignocellulosic biomass of aquatic macrophytes, the wall cell of *E. crassipes* is composed of:

*Lignin*, a hydrophobic amorphous heteropolymer, consisting of units of phenylpropene with hydroxyl and methoxy groups (phenyl group) that acts as a kind of cement between the fibrils and a stiffening agent between the fibers [52].

*Hemicellulose*, a hydrophilic polysaccharide, formed by polymerized monosaccharides, such as xylose and arabinose (carbohydrates with 5 carbons) and galactose, glucose, and mannose (carbohydrates with 6 carbons) [52].

*Cellulose,* a biopolymer formed by glucose molecules linked by β-1,4-glycosidic bonds containing six hydroxyl groups that establish interactions of intra and intermolecular hydrogen bonds that confer the crystallization capacity of cellulose making it insoluble in water [52, 53].

However, the concentration of those components varies from one plant to another of this species [52, 53]. Those three components (lignin, hemicellulose, cellulose) hold plant fibers together, offer mechanical protection, and act as a natural barrier against microbial action [52].

The chemical composition of the lignocellulosic structure of *E. crassipes* also includes potassium, calcium, magnesium, phosphorus, silica, carbon, and nitrogen whose quantities present in the biomass of this aquatic macrophyte depend strongly on its environment [53]. As a result of their overall composition explained above, the structure of *E. crassipes* has functional groups such as: –NH_2_ (amines), –COOH (carboxyls), –OH (hydroxyls), –SH (sulfhydryl), –C=O (carbonyls), among others involved in the removal of pollutants of the water [54, 55].

The main advantage of using *E. crassipes* as a biosorbent is the combination of low cost (due to its high availability) together with its effectiveness as a hyperaccumulator [38].

Many authors aiming to test the biosorption potential of *E. crassipes* have used the basic methylene blue dye and other commercial dyes as model molecules of contaminants [36–44].

#### *Eichhornia crassipes* as biosorbent of synthetic dyes

In recent years, the scientific community has studied the ability of *E. crassipes* to remove synthetic dyes in water. Some dyes and the respective parameters that influence the adsorption process are listed in Table 5.

**Table 5:** The adsorption capacity of dyes by *Eichhornia crassipes* and its respective operational parameters

Biosorbent	Adsorbate	pH	Temperature	*q*_max_	Reference
Dried roots	Methylene blue (C_16_H_18_ClN_3_S)	5–12	Room temperature	128.9 mg g^−1^	[56]
	Victoria blue B (C_33_H_32_ClN_3_)			145.4 mg g^−1^	
Dried roots	Methylene blue (C_16_H_18_ClN_3_S)	9.5	40 °C	42.55 mg g^−1^	[57]
	Malachite green (C_23_H_25_N_2_Cl)			44.64 mg g^−1^	
Activated carbon of stems	Methylene blue (C_16_H_18_ClN_3_S)	8.0		425.7 mg g^−1^	[58]
Activated carbon of leaves				275 mg g^−1^	
Dried stems				274 mg g^−1^	
Dried leaves				210.5 mg g^−1^	
Activated carbon of stems	Rhodamine B (C_28_H_31_ClN_2_O_3_)	Normal	298 K	280.2 mg g^−1^	
Activated carbon of leaves				156.5 mg g^−1^	
Dried stems				38.4 mg g^−1^	
Dried leaves				49.15 mg g^−1^	
Dried leaves and stems (treated with HNO_3_)	Methylene blue (C_16_H_18_N_3_Cl·3H_2_O)	3.7–4.4	27 °C	48.27 mg g^−1^	[59]
			45 °C	46.25 mg g^−1^	
			60 °C	47.10 mg g^−1^	
			80 °C	47.15 mg g^−1^	
Stems and leaves (not treated)	Methylene blue (C_16_H_18_N_3_Cl·3H_2_O)	< 6	Room temperature	254.5 mg g^−1^	[60]
Stems and leaves (thermally treated)		> 8		426.9 mg g^−1^	
Stems and leaves (treated with NaOH)		12		211.3 mg g^−1^	
Stems and leaves sulfonated		< 6		203.9 mg g^−1^	
Stems and leaves (treated with HCl)		< 6		198.0 mg g^−1^	
Dried roots	Rhodamine B (C_28_H_31_ClN_2_O_3_)	3–12	20 °C	27.15 mg g^−1^	[61]
Dried leaves				44.60 mg g^−1^	
Dried roots	Crystal violet (C_25_H_30_ClN_3_)	7.8	27 °C	322.58 mg g^−1^	[62]
Dried roots	Methylene blue (C_16_H_18_ClN_3_S)	8–10	30 °C	111.1 mg g^−1^	[63]
	Crystal Violet (C_25_H_30_ClN_3_)			43.5 mg g^−1^	
Entire plant	Congo red (C_32_H_232_N_6_Na_2_O_6_S_2_)	5.0	–	53.76 mg g^−1^	[64]
Living roots	Crystal violet (C_25_H_30_ClN_3_)	7.0	27 °C	20.84 mg g^−1^	[65]
Dried roots	Amaranth (C_20_H_11_N_2_O_10_S_3_Na_3_)	2.0	25 °C	28.51 mg g^−1^	[66]
Dried stems				23.97 mg g^−1^	
Dried leaves				43.1 mg g^−1^	
Entire plant				31.18 mg g^−1^	
Dried roots	Metanil yellow (C_18_H_14_N_3_NaO_3_S)	2.0	20 °C	30.27 mg g^−1^	[67]
Dried stems				27.5 mg g^−1^	
Dried leaves				43.5 mg g^−1^	
Entire plant				34.56 mg g^−1^	
Dried roots	BF−4B red reactive (C_31_H_19_N_7_Na_5_O_19_S_6_C)	2.0	30 °C	43.28 mg g^−1^	[68]
Leaves (not activated)	Safranin–O (C_20_H_19_N_4_Cl)	6.0	22 °C	88.7%	[69]
Leaves (thermally activated)		6.0		92.3%	
Leaves (acidly activated)		6.0		98.3%	

Low et al. [56] used dried roots of *E. crassipes* for the biosorption of Methylene Blue and Victoria Blue B. They demonstrated with their experiments that the roots of *E. crassipes* have a high removal potential of both basic dyes. The referred authors have shown that the removal of Methylene Blue and Victoria Blue B was unfavorable at a very low pH (< 2) and kept almost constant at higher pH (up to pH 12) (Table 5). Based on these results, the researchers suggested that at a very acid pH there is a competition between dye molecules and the excess of H^+^ ions by binding sites on the biosorbent surface. Experimental data were explained by the Langmuir isotherm, and the kinetic data were fitted to the Lagergren equation.

Saltabas et al. [57] applied dried roots of *E. crassipes* to the adsorption of the cationic dyes Methylene blue and Malachite green*.* Their experiments demonstrated that the removal of these dyes by the roots occurred quickly in 10 min. According to the authors, operational parameters such as pH of the solution and temperature of the system had a relevant influence on the biosorption of the dyes. Saltabas et al. [57] used the Langmuir and Freundlich equations to mathematically describe the biosorption equilibrium, but the Langmuir model was better to explain the equilibrium data obtained in the experiments. The thermodynamic parameters such as ∆G^o^, ∆H^o^, and ∆S^o^ were evaluated, and they concluded that the studied biosorption process was spontaneous and endothermic, and it was represented by the pseudo-second-order model.

El-wakil et al. [58] studied and compared the adsorption of Methylene Blue and Rhodamine B by the carbon of stems and leaves of *E. Crassipes*, and the carbon materials were activated by H_3_PO_4_. According to the authors, operational parameters such as the initial concentration of dyes, temperature, and pH of the aqueous solutions influenced the adsorption of Methylene Blue and Rhodamine B. Their results also suggested that a decrease in adsorption at low pH may have occurred due to electrostatic repulsion and competition between H^+^ ions and the cationic dyes, by the binding sites positively charged on the biosorbent surface. At high pH there was an increase in the number of negatively charged binding sites, favoring the removal of Methylene Blue and Rhodamine B by the surfaces of the dry biomass and the activated carbon of *Eichornia crasssipes.* The adsorption equilibrium data were fitted to the isothermic Langmuir model, and the kinetic model followed was the pseudo-second-order.

El-Khaiary et al. [59] studied the biosorption of Methylene Blue using dried leaves and stems of *E.* crassipes chemically treated by HNO_3_. According to the authors, the adsorption of Methylene blue was influenced by initial dye concentration, the temperature of the system, and contact time (between the biosorbent and adsorbate). They reported that the kinetic pseudo-second-order model was better fitted to the results obtained in the experiments conducted at room temperature; however, both the pseudo-second-order model as the Lagergren isotherm was better fitted to the results obtained at high temperatures (45–80 °C).

In a subsequent study, El-Khaiary et al. [60] analyzed again the removal of Methylene Blue applying dried leaves and stems of *E. crassipes* sulfonated and chemically treated by immersion in HNO_3_, NaOH, and HCl solutions. In this study, the influence of pH and the initial concentration of Methylene blue on the biosorption process were studied. According to the authors, the Langmuir isotherm well represents the adsorption data obtained by the biosorbents chemically treated, thus suggesting a surface energetically homogeneous in the biosorbent, except for the biomass immersed in NaOH whose data were best represented by the Freundlich isotherm.

Saufi et al. [61] investigated the biosorption of Rhodamine B by the roots and dried leaves of *E. crassipes.* They used Fourier transform infrared spectroscopy (FTIR), X-ray diffraction (XRD), scanning electron microscopy (SEM), and thermogravimetric analysis (TGA) to analyze the characteristics of the biosorbents. According to the report by the authors, the leaves showed a higher biosorption capacity of Rhodamine B than the roots, and the increase of initial dye concentrations along with the contact time (between biosorbent and dye solution) intensified the dye removal by the roots and leaves of the plant. Saufi et al. [61] concluded that the initial increase of biosorbent concentrations of 5 mg/L to 60 mg/L and contact time contributed to the elevation of the dye adsorption by *E. crassipes.* The pseudo-second-order equations well represented the experimental kinetics data, and the Langmuir model best represented the dye adsorption isotherm data.

Kulkarni et al. [62] studied the biosorption capacity of dried roots of *E. crassipes* using crystal violet dye as the adsorbate. Kulkarni et al. [62] examined the influence of parameters such as pH of the dye solution, dosage of the biosorbent, initial concentration of crystal violet, and the temperature of the system on the biosorption process. The authors suggested that at high temperatures the bond between the dye molecules and the bonding sites existing in the roots of *E. crassipes* (sprayed) becomes more intense. The Langmuir and Freundlich isotherm models were tested, and the Freundlich model better described the biosorption equilibrium data, while the pseudo-second-order model best represented the biosorption kinetics data.

Mahamadi and Mawere [63] examined the removal of two basic dyes Methylene Blue and Crystal Violet from aqueous solutions in monocomponent and binary systems by the dried roots of *E. crassipes* treated with HNO_3_ and immobilized on alginate beads. In this study, the roots of *E. crassipes* showed greater affinity for Methylene Blue molecules than for Crystal Violet in both monocomponent and binary systems. According to Mahamadi and Mawere [63], the mathematical Langmuir model was the most suitable to describe the adsorption equilibrium data suggesting therefore that the dye biosorption on the surface of biosorbent occurred in monolayers.

Parvin et al. [64] show in their studies that the dried biomass of *E. crassipes* has the potential to remove Congo Red dye from wastewater. The studies led by the authors took into account the influence of parameters such as pH, the dosage of biosorbent, initial concentration of dye, and contact time. According to the authors, the Langmuir isotherm model has proved to be more adequate to describe the biosorption data and suggested that the process occurred in monolayers on the surface of the biosorbent. Parvin et al*.* [64] concluded, based on the results obtained in the studies of biosorption kinetic, followed by the pseudo-second-order models and the intraparticle diffusion model, that there was a very notable biosorption action on the surface of the biomass of *E. crassipes.*

Patil et al. [65] investigated the potential for biosorption of live roots of *E. crassipes* to remove the cationic dye crystal violet from aqueous solutions. The authors tested the isotherm models of Dubinin–Radushkevich, Temkin, Freundlich, and Langmuir to elucidate the mechanisms that act in the biosorption of the dye. Among the mathematical models used to describe the biosorption data, the isotherm Freundlich was more appropriate (with R^2^ = 0,999), suggesting that the biosorption process occurred in multilayers on an energetically heterogeneous surface. Patil et al*.* [65] observed the influence of contact time on the crystal violet biosorption performed by *E. crassipes*. In this study, the pseudo-second-order model was the most adequate to describe the adsorption equilibrium.

Guerrero-coronilla et al. [66] studied the biosorption of the Amaranth dye by whole *E. crassipes* and by their dried roots, leaves, and stems (separately). They proved that the leaves performed well the dye biosorption. According to the researchers, the results obtained by FTIR confirmed that functional groups –OH, C=O, and NH present in proteins and polysaccharides of the plant participated actively in the removal of the dye. The FTIR analysis indicated a strong interaction between the proteins present in *E. crassipes* biomass, such as starches I and II, with the Amaranth molecules, particularly in the leaves. Experimental data were well represented by the pseudo-second-order model.

Subsequently, in a similar study, Guerrero-Coronilla et al. [67] explored the capacity of the anionic dye to remove Metanil Yellow by dried entire *E. crassipes* and its dried vegetative organs separated (roots, stems, and leaves). Guerrero-Coronilla et al. [67] concluded that proteins played a fundamental role in the biosorption process and that the dried leaves of *E. crassipes* had a better performance in removing Metanil Yellow than the rest of the plant, corroborating with their results obtained in the previous works [66]. The presence of Metanil Yellow adsorbed on the surface of the biosorbent was proved by scanning electron microscopy (SEM) and scanning confocal microscopy laser (CLSM) examination.

Rigueto et al. [68] evaluated the potential of dry roots of *E. crassipes *to remove the reactive Red dye BF-4B from an aqueous solution. Analysis by scanning electron microscopy (SEM) made by the researchers showed that there were no chemical changes on the surface of the biosorbent before or after the biosorption process. The same authors proved that the highest rate of dye removal by the roots (of the plant) occurred at acidic pH and by the increase of the system temperature. The pseudo-second-order and Elovich models were adequate to represent the profiles of the kinetics studies, while the biosorption equilibrium data were well represented by the Langmuir isotherm.

Mohammed et al. [69] tested the removal of Safranin–O dye from aqueous solutions by dried leaves of *E. crassipes:* without any activation, thermally activated, and chemically activated by HCl. The authors examined the roots before and after experiments using the Fourier transform infrared spectroscopy (FTIR) analysis and observed the presence of –OH groups in the biosorbent structure. Mohammed et al*.* [69] reported that the roots subjected to chemical treatment with HCl showed a better performance in the adsorption of Safranin–O than the untreated and thermally treated roots.

#### *Eichhornia crassipes* as biosorbents of heavy metal cations

The effect of different parameters on the adsorption of a variety of metallic species (mainly heavy metals) by the biomass of *E. crassipes,* in mono- or multimetallic aqueous systems, has been studied by several authors, as shown in Table 6.

**Table 6:** The adsorption capacity of metals by *Eichhornia crassipes* and its respective operational parameters

Biosorbent	Adsobate	pH	Temperature	*q*_max_	Reference
Dried roots	Pb	4.8	30 °C	26.32 mg g^−1^	[70]
	Pb–Cd			25.38 mg g^−1^	
	Pb–Zn			22.12 mg g^−1^	
	Pb–Zn–Cd			14.31 mg g^−1^	
	Cd			12.59 mg g^−1^	
	Cd–Zn			Not fitted	
	Cd–Zn–Pb			3.04 mg g^−1^	
	Zn			12.55 mg g^−1^	
	Zn–Pb			4.32 mg g^−1^	
	Zn–Cd			Not fitted	
	Zn–Pb–Cd			3.66 mg g^−1^	
*E. crassipes* ashes	Pb	> 8	25 °C	29.83 µg g^−1^	[38]
	Cr	> 8		24.00 µg g^−1^	
	Zn			29.94 µg g^−1^	
	Cd			28.41 µg g^−1^	
	Cu			29.83 µg g^−1^	
	Ni			29.79 µg g^−1^	
Dried stems and leaves	Cu	4.5	25 °C	27.7 mg g^−1^	[71]
Dried roots	Cd	6.0	45 °C	104 mg g^−1^	[72]
Dried roots	U	5.0	25 °C	64.000 µg g^−1^	[73]
Dried stems and leaves	U	5.5	45 °C	142,85 mg g^−1^	[74]
Entire plant	Pb	5.0	25 °C	75.44 mg g^−1^	[75]
Dried roots	Co	8.0	25 °C	86.9%	[76]
Activated carbon of *E. crassipes*	Hg	5.0	30 °C	28.40 mg g^−1^	[77]
Dried shoots of *E. crassipes*	Pb	5.0	25 °C	92.90%	[78]
	Cd			79.22%	
Dried roots	Pb			94.02%	
	Cd			79.65%	
Dried roots	Pb	5.0	–	87.61 mg g^−1^	[79]
	Cd	5.0		66.16 mg g^−1^	
	Zn	5.0		70.23 mg g^−1^	
	Cu	6.0	–	35.62 mg g^−1^	

Mahamadi and Nharingo [70] studied the competitive adsorption of ions Pb^2+^, Cd^2+^, and Zn^2+^ in single, binary, and ternary systems on dried roots of *E. crassipes* treated with HNO_3_. They observed that the ions of Pb^2+^ were satisfactorily removed from the aqueous medium in the presence of Cd^2+^ and Zn^2+^ (separately, in the following order: Pb > Cd, Pb > Zn), while the combination of the three metals in the system suppressed the removal of Cd^2+^ and Zn^2+^ in the presence of Pb^2+^ (Pb >  > Cd > Zn). The removal of Cd^2+^ and Zn^2+^ in monocomponent and binary systems showed a greater potential of adsorption in absence of Pb^2+^. The competitive Langmuir model proved to be well adjusted to the analysis of the binary (except for Zn–Cd, and Cd–Zn combinations) and ternary equilibrium sorption data presented in the study. All removals reported in single, binary, and ternary systems can be observed in Table 6.

Mahmood et al*.* [38] tested the adsorptive ability of *E. crassipes* ashes in the biosorption of Pb^2+^, Cr^6+^, Zn^2+^, Cd^2+^, Cu^2+^, and Ni^2+^ in aqueous solutions. Results obtained by the authors using X-ray diffraction (XRD) showed the presence of oxides such as Ca, Mg, K, Na, Al, and Si in ashes of *E. crassipes*. They suggested that these oxides when reacted with the aqueous medium, produced hydroxides that increased the pH and intensified the adsorption of the metallic ions by the ashes of *E. crassipes*. According to the authors, the process may have occurred mediated by ion exchange mechanisms. Mahmood et al*.*[38] concluded that the ashes derived from *E. crassipes* satisfied the criteria of a hyperaccumulator of Pb^2+^, Zn^2+^, Cu^2+^, Cr^6+^, Zn^2+^, and Ni^2+^.

Komy et al*.*[71] evaluated the accumulation of Cu^2+^ by leaves and dried stems of *E. crassipes* and proved through analyses made by Fourier transform infrared spectroscopy (FTIR) that the plant contains functional groups such as –OH, –C=O, P=O, and COOH which act as the main binding sites of Cu^2+^. According to Komy et al*.*[71], the groups -OH were found in abundant quantity in the plant and can be one of the main responsible for adsorption of Cu^2+^ by *E. crassipes*, by mechanisms as chelation. The maximum adsorption capacity and Langmuir isotherm constants were evaluated at 3 different pHs using the Pardo and Norton models, which showed a better result at pH 4.5 (Table 2).

Murithi et al*.*[72] concluded in their studies that the adsorption of Cd^2+^ by dried roots of *E. crassipes* was highly dependent on operational parameters such as pH, temperature, initial concentration of metal ions, and magnetic stirring speed. The authors studied the influence of Na^+^, K^+^, Mg^2+^, and Ca^2+^ ions on the adsorption of Cd^2+^ and reported that the presence of Na^+^ and K^+^ did not affect the biosorption process significantly, unlike the Mg^2+^ and Ca^2+^ ions, which caused a considerable decrease in the metal retention. Murithi et al*.*[72] concluded that the roots of *E. crassipes* have a greater capacity for sequestering Cd^2+^ from aqueous solutions than other biosorbents used to remove this metal. The biosorption equilibrium was described by the Freundlich and Langmuir isotherms, and the Langmuir model was better adjusted to the experimental data than the Freundlich model.

Shawky et al. [73] examined the biosorption potential of dried roots of *E. crassipes* to remove U^6+^. According to Shawky et al. [73], the biosorption process U^6+^ was influenced by factors such as pH, initial concentration of U^6+^ in the solution, dried roots weight, and contact time between roots and U^6+^ solution. The tests were carried out with five different biosorbent dosages (20, 40, 60, 80, and 100 µg mL^−1^) and related that the maximum adsorption U^6+^ by *E. crassipes* occurred rapidly, after 10-min contact time between the biosorbent and the adsorbate. The authors reported that U^6+^ adsorption followed the Langmuir isotherms and that the favorable biosorption of U^6+^ in monolayers occurred due to the mutual attraction between positively charged ions and the negatively charged root surface.

Yi et al. [74] investigated the biosorption of U^6+^ from aqueous solutions by dried biomass (stems and leaves) of *E. crassipes*. The experiments showed that U^6+^ biosorption was highly dependent on pH and that mechanisms such as complexation and electrostatic attraction participated actively in the biosorption process. The analyses carried out by Yi et al. [74] using Fourier transform infrared spectroscopy (FTIR) and X-ray photoelectron spectroscopy analysis (XPS) indicated that functional groups containing amine (–NH_2_), hydroxyl (–OH), and carboxyl (–COOH) were involved in the biosorption of U^6+^. The authors showed that the pH of the solution had a significant influence on the adsorption of U^6+^ by *E. crassipes.* The metal adsorption increased dramatically with the increase of pH and declined with a pH above 5.5. The phenomenon was explained by the difference in the content of various U^6+^ species at different pH values. At very low pH, the functional groups (NH_2_, OH, and COOH) on the dried biomass of *E. crassipes* surface are protonated, whereas positively charged uranium exists mainly in the form of uranyl cations, which are hardly captured. Contrary, at relatively high solution pH, the functional groups became deprotonated, which led to an increase of the negative charge of the *E. crassipes* surface and favored the binding of positively charged uranium species. However, as reported by Yi et al. [74], the continuous increase of pH above 5.5 resulted in a decrease in the biosorption of U^6+^ in aqueous media. Above pH 5.5, the schoepite precipitate (4UO_3_.9H_2_O), thus resulting in the decline of the U(VI) removal efficiency. Yi et al. [74] proved that the biosorption of the metal by the biomass of *E. crassipes* took place in a contact time of approximately 10 min, similar to what occurred in the experiments previously carried out by Shawky et al. [73].

In the experiments carried out later by Yi et al. [75], they studied the feasibility of using the dried biomass of *E. crassipes* for the removal of Pb^2+^ from liquid effluents. The experiments performed by Yi et al. [75] demonstrated that the Pb^2+^ adsorption process is highly dependent on the pH of the medium. According to the authors, at a pH above 6.0, there was the formation of Pb^2+^ hydroxides that tended to precipitate when the pH increased. Yi et al. [75] examined the morphology of *E. crassipes* grains before and after contact with Pb^2+^ ions and in these experiments were able to observe via scanning electron microscopy (SEM) that streaks with different widths were found in a row on the surface of *E. crassipes* granules after exposure to Pb^2+^. These results also suggested that the irregular surface of depressions found in the biosorbent could aid in the adsorption of Pb^2+^ and contribute to larger adsorption of Pb^2+^ on the surface of the biosorbent.

Arafat [76] examined the biosorption potential of dried *E. crassipes* roots in removing Co^2+^ from aqueous solutions. The author reported that the removal of Co^2+^ by the roots was also highly dependent on factors such as the concentration of Co^2+^, roots weight, and pH of the solution. The results obtained by Arafat [76] demonstrated that maximum adsorption of Co^2+^ occurred after 10 min, having a similar result to that obtained in biosorption experiments U^6+^ for *E. crassipes* roots performed by Shawky et al*.* [73]. The author concluded that there was high retention of Co^2+^ ions (from 52.6 to 86.9%) by the increase of weight of *E. crassipes *roots from 0.02 to 0.1 g, respectively. Additionally, the amount of Co^2+^ removed widely increased (from 10.8% to 86.9%) by increasing the pH of the solution (from 2 to 8).

Kadirvelu et al. [77] investigated the adsorption and desorption of Hg^2+^ by activated carbon derived from *E. crassipes* with a granulometric range in the order of 125–180 µm. According to the researchers, the biosorption Hg^2+^ by *E. crassipes* as activated carbon was dependent on the pH of the system, carbon dosage, and initial concentration of Hg^2+^. Kadirvelu et al. [77] found that active carbon and ions Hg^2+^, positively charged, compete with protons H^+^ for binding sites existing on the surface of the activated carbon at acid pH, leading to a decline in the adsorption of Hg^2+^. On the other hand, with the increase of pH, the concentration of H^+^ tended to decrease, causing a consequent increase in the removal of metal by the activated carbon. Kadirvelu et al. [77] used HCl and KI solutions in the desorption tests and obtained a Hg^2+^ desorption rate of 15% and 30%, respectively. The kinetic models of first and second order proved to be well suitable for the biosorption kinetic tests, while the biosorption isotherm data were well represented by both the models Freundlich and Langmuir.

Ibrahim et al. [78] evaluated the adsorption of Cd^2+^ and Pb^2+^ ions by dried shoots and roots of *E. crassipes* in aqueous solutions. The kinetic experiments demonstrated that the biosorption equilibrium was achieved in a rapid interval of time, approximately from 30 to 60 min. According to Ibrahim et al. [78], the roots and shoots of *E. crassipes* show similar ability on the removal of Cd^2+^ and Pb^2+^. Ibrahim et al. [78] concluded through Fourier transform infrared spectroscopy (FTIR) analysis that hydrogen bonds present in functional groups like –COOH mediated the removal of the cations by both the parts of the plant. The data of biosorption kinetics were well described applying the second-order model.

Li et al. [79] conducted experiments applying dried roots of *E. crassipes* as biosorbent of Pb, Zn, Cu, and Cd in aqueous medium. Li et al. [79] investigated the influence of parameters, such as biosorbent and adsorbate concentration, temperature, pH in monometallic and multi-metallic solutions. The results obtained by the authors showed that the adsorption followed the order of Pb > Cd > Zn > Cu, as such the removal of Cd and Zn was inhibited for the presence of Cu and P. Based on Fourier transform infrared spectroscopy (FTIR) analysis, Li et al.[79] found the presence of carboxyl, phosphorous, and nitrogenous compounds in the roots and suggested that those functional groups are directly involved in the process of biosorption. The morphological characteristics and the properties of the beads of dried roots were characterized by scanning electron microscopy (SEM) and surface area (SBET). The Freundlich isotherm model and second-order kinetics model elucidated well the biosorption of Pb, Zn, Cu, and Cd in mono- and multimetallic systems by the roots of *E. crassipes.* Table 6 shows a set of results of biosorption of metal ions by *E. crassipes.*

#### Biosorption mechanism

There are two major differences between using live macrophytes (which lead to the phytoremediation process) and dead macrophytes (or inactive plant which leads to the biosorption process).

*In live macrophytes*, the absorption of metals such as Cr, Co, Cu, Mn, and Zn is essential for their physiological and metabolic functions; however, these chemical species can be toxic when present in high concentrations in plant tissues [80, 81]. The excess of heavy metals inside plant cells (accumulation) can compromise the functions of the plasma membrane and lead to the formation of free radicals and reactive oxygen species, such as OH^−^, O^2–^, and H_2_O_2_, resulting in oxidative stress [81, 82]. Some studies suggest that the hyperaccumulation of these metals in some plant species is a defense mechanism against natural enemies such as microbial pathogens and herbivorous animals [80]. Among tolerant and hyperaccumulating plants, *E. crassipes* can be highlighted, whose ability to survive in highly contaminated aquatic environments may be associated with the fact that its roots are full of micropores and have active centers (composed by functional groups as detailed in Table 3) that favor the binding and the ion exchange of bivalent and polyvalent cations [81]. The cell walls of the root tissues of these plants are externally charged with these functional groups (which can be negatively charged or fully protonated), which adsorb these metals in water, thus acting as initial barriers [83].

The high affinity of the ions of these metals for the actives centers restricts access to vascular systems and, consequently, the translocation and concentration of these metals in the aerial parts of plants [84]. The passage of these metals is also blocked by apoplastic barriers such as Caspary's striae, located in the primary root absorption region (in the endoderm), which keeps a part of them concentrated in the roots [82].

It should be noted that several mechanisms work together to reduce toxicity caused by toxic metal species [82]. These processes reduce the concentration of these metals in the plant's cytosol and organelles, thereby preventing the denaturation of enzymes essential to the metabolism of plants and the reduction of their metabolic activities [85].

*In dead macrophytes,* their mechanisms of metal and dye removal by biosorption can be classified as extracellular accumulation/precipitation, cell surface sorption/precipitation, and intracellular accumulation as described for freshwater macrophytes such as *Salvinia hergozi, Potamogeton lucens, Eichhornia crassipes, Cabomba sp.; Myriophyllum spicatum*, and *Ceratophyllum demersum* who have been investigated previously [86–89]. The dead macrophytes have been tested in biosorption in their modified form such as dried root, activated carbon, biochar, and ash (for example derived from *Eicchornia Crassipes*), soft chemical treatments (such acid-/alkali-treatment). A set of articles that used died macrophyte *Eicchornia Crassipes* is shown in Tables 5 and 6.

Diverse functional groups such as carboxyl, hydroxyl, sulfate, and amino groups (see Table 3) which are originally present on the biosorbent are responsible for possible binding mechanisms in the dead microphytes. The contaminants in the aqueous solution bind through those functional groups on the biosorbent surfaces at particular pH, and precipitation occurs. The biosorption is influenced by pH of the solution, temperature, quantity of biomass, concentration of contaminants, surface area, and particle porosity and tortuosity (these both affect the pore diffusion) [89].

Additionally, it must be considered that the adsorption ability of the surface and the surface-active centers is influenced by the point of zero charge (pH_pzc_), as well. Even though the value of pH is used to describe pzc only for systems in which H^+^/OH^−^ are the potential determining ions, it may help to explain the biosorption of cationic and anionic dyes, as well as metallic ions in water. For example, in the presence of functional group such as OH− group on surface, cationic dye adsorption is favored at pH > pH_pzc_, whereas anionic dye adsorption is favored at pH < pH_pzc_ where the surface becomes positively charged. The value pH_pzc_ of biosorbents is an important property to understand the adsorption mechanism [90].

Up to the date of the publication of this article, there were not any chemical mechanisms proposed for the biosorption of metal and synthetic dyes.

Additionally, it must be considered that in the biosorption processes the adsorption kinetics controls the overall rate, which determines the time required for reaching equilibrium for the adsorption process. At the solid–liquid interface, the pathway of adsorption can be described by the following steps [91]:

1. Bulk diffusion (the adsorbate is transported from the bulk of the solution to the liquid film surrounding the adsorbent).
2. External diffusion (the adsorbate diffuses across the surface liquid film surrounding the adsorbent).
3. Intraparticle diffusion of the adsorbate from the liquid film to the surface of the solid adsorbent particles. This step occurs through two mechanisms: (a) pore diffusion and (b) surface diffusion.
4. Interaction with the surface-active sites (either by chemisorption or physisorption). In the case of reversible adsorption, it must also have to include the desorption of the adsorbate.

The overall rate of the adsorption process will be controlled by the slowest steps.

The various kinetic models such as pseudo-first-order, pseudo-second-order, Elovich, and Weber's intraparticle diffusion are generally used to understand the detailed characteristics of the adsorption process. In these models, the kinetic constants of adsorption are calculated, and to evaluate the best fit model the linear regression correlation coefficient (*R*^2^) values are compared.

The kinetic mechanism of adsorption of Methylene blue dye in aqueous solution by *E. crassipes* is usually described as a pseudo-second-order model (for example [88] reported 15 min at room temperature, which makes the process practical for industrial application).

Adsorption isotherms describe how different adsorbates interact with an adsorbent, through the correlation between the two parameters, *q*_e_ and *C*_e_ (at constant temperature and pH).

There are many isotherm models used to extract preliminary information about the adsorption mechanisms. Isotherm models were used over the years, such as Langmuir, Freundlich, Redlich–Peterson, Dubinin–Radushkevich among others (some with theoretical foundation and others being of empirical nature) [91]. The Langmuir and Freundlich models are the most commonly used for the description of adsorption data.

## Conclusions and future perspectives

The whole *E. crassipes* and its parts (roots, stems, and leaves), in natural form and carbon derived, have been investigated in the removal of contaminants (dyes and metals) from aqueous solutions. All results suggest that *E. crassipes* and carbon derived are promising for wastewater treatment.

During the last 27 years, 333 documents reported the efficiency of *E. crassipes* in the removal of a wide variety of pollutants. The operational parameters such as pH, temperature, dosage, and contact time to reach the best conditions for the removal of many pollutants (special synthetic dyes and heavy metals) are well-established and known. However, the exact chemical mechanism of biosorption of *E. crassipes* (dead biomass) in the removal of heavy metals and dye is still unknown.

Together with that, scale-up studies, adsorption in continuous systems, diffusional effects, regeneration, and economic studies deserve more attention as these issues are very important to enable the use of *E. crassipes* in wastewater systems and drinking water treatment plants. Moreover, once *E. crassipes* be well studied, the biosorbent could be used in the manufacturing of biofilters which could be available to poor communities that live in places where basic sanitation does not exist.

Carbon derived from *E. crassipes* is very attractive, since *E. crassipes* is widely available in many countries, renewable, and not cost. The carbon derived is, by far, a better adsorbent than the natural *E. crassipes*; however, the carbon production requires high energy demand; therefore, comparison studies are needed to evaluate the balance between economic advantages and efficiency in adsorption.

The possibility of the utilization of *E. crassipes* in treatments of contaminated water, more specifically from industry, as a viable and eco-friendly alternative is strongly suggested. We believe that *E. crassipes* has so much to contribute to the scientific community and society. In particular, in issues concerning Green Chemistry Principles, Sustainable Development Goals (United Nations), and the current situation of the increasing poverty worldwide (that includes the water needs) triggered by the current pandemic COVID-19. To reach Sustainable Development Goals more sustainable, economic, and effective processes for water treatment are needed; in this scene, *E. crassipes* seem to be a very useful tool to reach these goals.
